# Synthesis and crystal structure of *catena*-poly[[bis[(2,2′;6′,2′′-terpyridine)­manganese(II)]-μ_4_-penta­thio­dianti­monato] tetra­hydrate] showing a 1D MnSbS network

**DOI:** 10.1107/S2056989019016268

**Published:** 2020-01-01

**Authors:** Felix Danker, Christian Näther, Wolfgang Bensch

**Affiliations:** a Institut für Anorganische Chemie, Universität Kiel, Max-Eyth. Str. 2, 241128 Kiel, Germany

**Keywords:** crystal structure,thio­anti­monate, chain compound, hydrogen bonding

## Abstract

The asymmetric unit of the title compound, {[Mn_2_Sb_2_S_5_(C_15_H_11_N_3_)_2_]·4H_2_O}_*n*_, consists of two crystallographically independent Mn^II^ ions, two unique terpyridine ligands, one [Sb_2_S_5_]^4−^ anion and four solvent water mol­ecules, all of which are located in general positions. The [Sb_2_S_5_]^4−^ anion consists of two SbS_3_ units that share common corners. Each of the Mn^II^ ions is fivefold coordinated by two symmetry-related S atoms of [Sb_2_S_5_]^4−^ anions and three N atoms of a terpyridine ligand within an irregular coordination. Each two anions are linked by two [Mn(terpyridine)]^2+^ cations into chains along the *c*-axis direction that consist of eight-membered Mn_2_Sb_2_S4 rings. These chains are further connected into a three-dimensional network by inter­molecular O—H⋯O and O—H⋯S hydrogen bonds. The crystal investigated was twinned and therefore, a twin refinement using data in HKLF-5 [Sheldrick (2015 ▸). *Acta Cryst.* C**71**, 3–8] format was performed.

## Abstract 2

In the crystal structure of the title compound, two [Sb_2_S_5_ [anions built up of two SbS_3_ units sharing common corners with each linked by two [Mn(terpyridine)]^2+^ cations into chains that are further linked into a 3D network by inter­molecular O—H⋯O and O—H⋯S hydrogen bonding.

## Chemical context   

Inorganic–organic chalcogenidometallates are an important class of compounds that have been systematically investigated for several decades (Sheldrick & Wachhold, 1998 ▸; Dehnen & Melullis, 2007 ▸; Zhou *et al.*, 2009 ▸; Seidlhofer *et al.*, 2010 ▸; Wang *et al.*, 2016 ▸; Zhou, 2016 ▸; Zhu & Dai, 2017 ▸). Therefore, a variety of compounds have been reported and some of them have potential for applications in different fields (Seidlhofer *et al.*, 2011 ▸; Nie *et al.*, 2014 ▸, 2016 ▸, 2017 ▸; Yue *et al.*; 2014 ▸). In this context, thio­anti­monates and thio­selenates are of special inter­est because they consist of primary building units that show a variety of coordination numbers, which can be traced back to the lone electron pair of anti­mony (Bensch *et al.*, 1997 ▸; Spetzler *et al.*, 2004 ▸; Stähler *et al.*, 2001 ▸; Lühmann *et al.*, 2008 ▸). These primary building units can be further linked into discrete anions or networks of different dimensionality (Jia *et al.*, 2004 ▸; Powell *et al.*, 2005 ▸; Zhang *et al.*, 2007 ▸; Liu & Zhou, 2011 ▸). This is the main reason why we have been inter­ested in this class of compounds for many years.

In the course of these investigations we have prepared compounds with the general composition Mn_2_ *L*Sb_2_S_5_ or Mn_2_ *L*_2_Sb_2_S_5_ with *L* as an mono-coordinating or a bis-chelating amine ligand such as, for example, methyl­amine, ethyl­amine, ethyl­enedi­amine or 1,3-di­amino­propane (Bensch & Schur, 1996 ▸; Schur & Bensch, 2002 ▸; Schur *et al.*, 2001 ▸). All of these compounds consist of SbS_3_ pyramids as primary building units as well as MnS_6_ and MnS_4_N_2_ distorted octa­hedra. These units are linked to form Mn_2_Sb_2_S_4_ hetero-cubane-like units that share common corners, edges and faces with a neighbouring heterocubane unit. These secondary building units are inter­connected into layers. Within the MnSbS network, the SbS_3_ pyramids are linked *via* common edges into chains. Thus, no discrete [Sb_2_S_5_]^4−^ anions are present. The N atoms of the amine ligands in these compounds are coordinated to the Mn^II^ ions and are always in the *cis*-position, thus arranged to form extended networks *via* Mn—S bond formation. Similar compounds have also been reported with 1,3-di­amino­pentane, di­ethyl­enetri­amine and *N*-methyl-1,3-di­amino­propane as ligands (Puls *et al.*, 2006 ▸; Engelke *et al.*, 2004 ▸). It is noted that di­ethyl­enetri­amine acts as a bis-chelating ligand, because the central N atom is not involved in the Mn coordination.

To reduce the dimensionality of the MnSbS network that might allow access to discrete [Sb_2_S_5_]^4−^ anions, we used the tetra­dentate ligand tris­(2-amino­eth­yl)amine for the synthesis of such MnSbS compounds. In this case, a compound with the composition Mn_2_(tris­(2-amino­eth­yl)amine)_2_Sb_2_S_5_ was obtained, in which all four N atoms of the amine ligand are involved in the Mn coordination (Schaefer *et al.*, 2004 ▸). In this case, only two of the six coordination sites of the Mn^II^ cations are accessible for Mn—S bond formation. This compound consists of discrete [Sb_2_S_5_]^4−^ anions, in which two SbS_3_ pyramids are joined together *via* a common sulfur atom, which is in contrast to the compound mentioned above, where the SbS_3_ units are linked by common sulfur edges into chains. These anions are connected to two [Mn(tris­(2-amino­eth­yl)amine)]^2+^ cations *via* the *cis*-coordinating terminal S atoms, forming discrete units instead of the condensed networks with mono-coordinating or bis-chelating ligands.
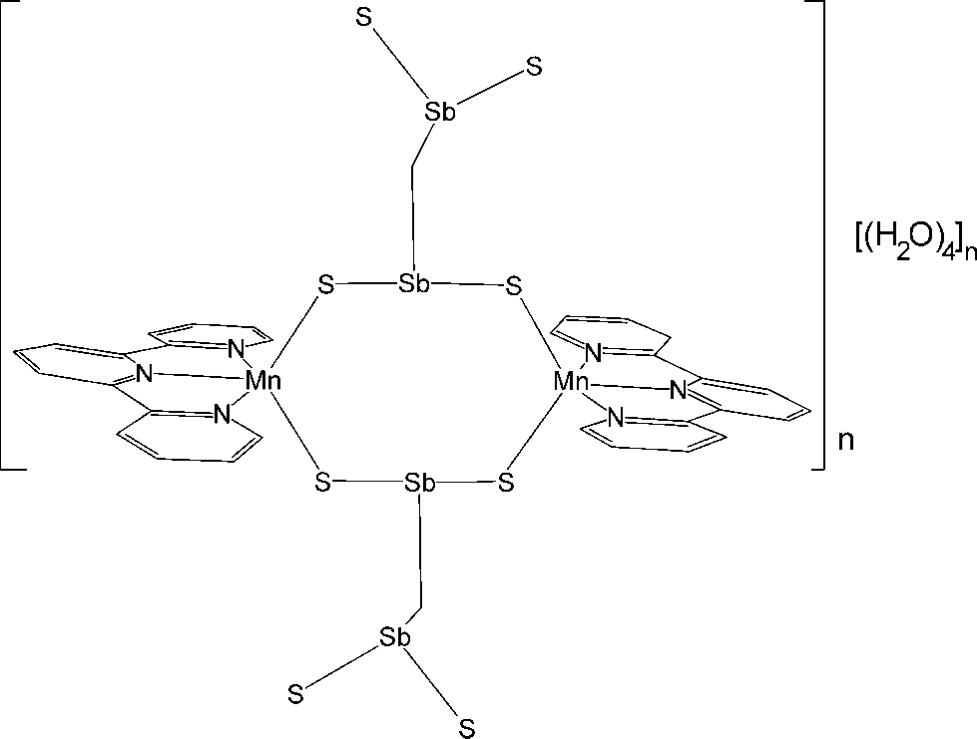


Based on these results, the question arose as to what kind of compound would be obtained with a tris-chelate ligand, in which all three N atoms are coordinated to the Mn^II^ ions but no such compound was obtained. In this context it is noted that all of these thio­anti­monates were prepared under solvothermal conditions using the elements as educts, but in future work we developed an alternative synthetic route using Na_3_SbS_3_ as reactant, for which the synthesis of such compounds is easier. Therefore, the tris-chelating ligand 2,2′;6′,2′′-terpyridine was reacted with Na_3_SbS_3_, leading to the formation of a new manganese thio­anti­monate with the composition Mn_2_(terpyridine)_2_Sb_2_S_5_^.^4(H_2_O) in which discrete [Sb_2_S_5_]^4−^ anions are present that link the [Mn(terpyridine)]^2+^ cations into a one-dimensional MnSbS network. X-ray powder measurements prove that the major phase consists of the title compound, but that some amorphous and a very small amount of an unknown crystalline phase is present (see Fig. S1 in the supporting information). This compound decomposes on storage, presumably because of the loss of the water mol­ecules.

## Structural commentary   

**Figure 1:**
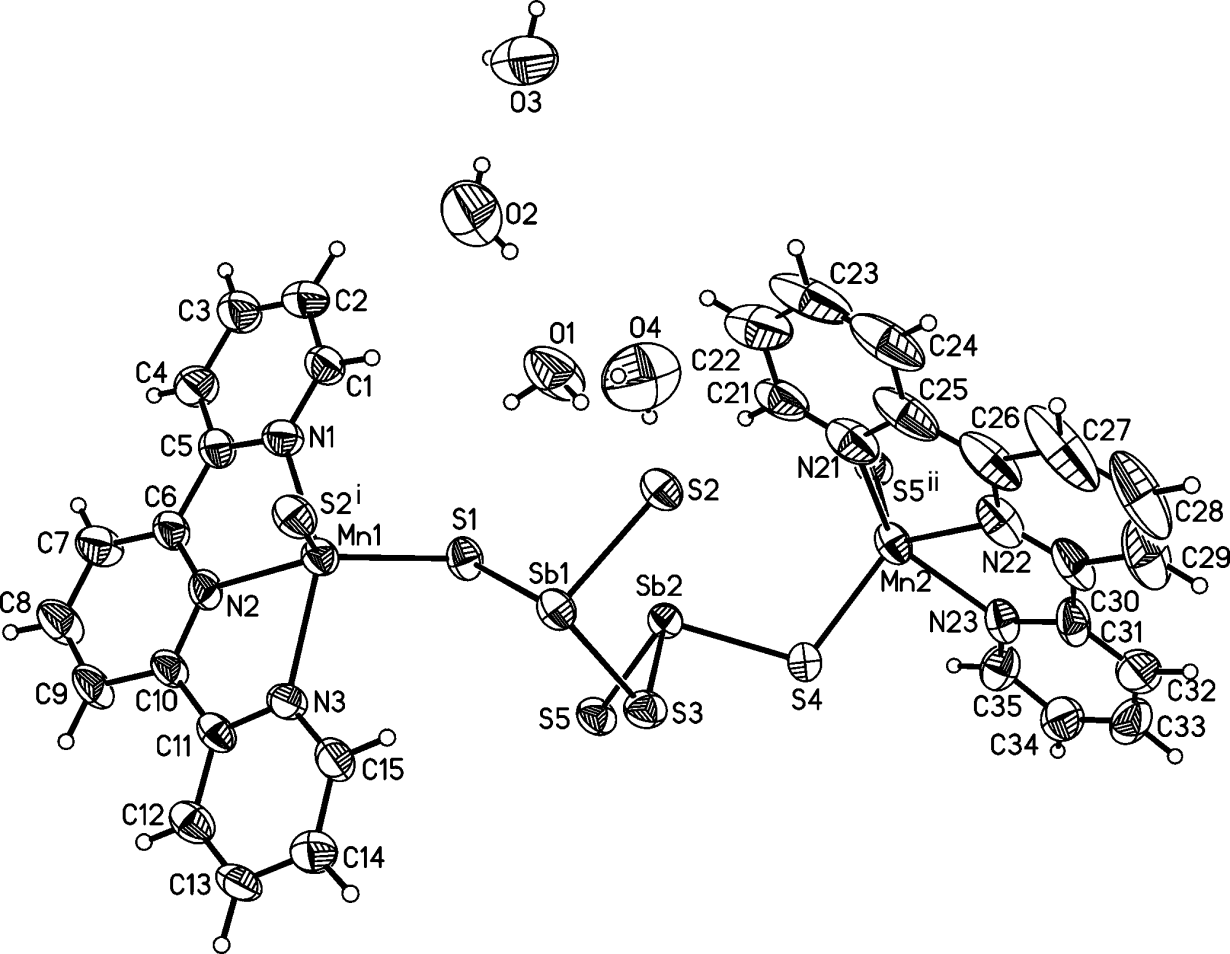
The asymmetric unit of the title compound with the atom-labelling scheme and displacement ellipsoids drawn at the 50% probability level. Symmetry-related atoms are included to complete the coordination of the Mn^II^ ions [symmetry codes: (i) −*x* + 1, −*y* + 1, −*z* + 1; (ii) −*x* + 1, −*y* + 1, −*z* + 2].

**Figure 2:**
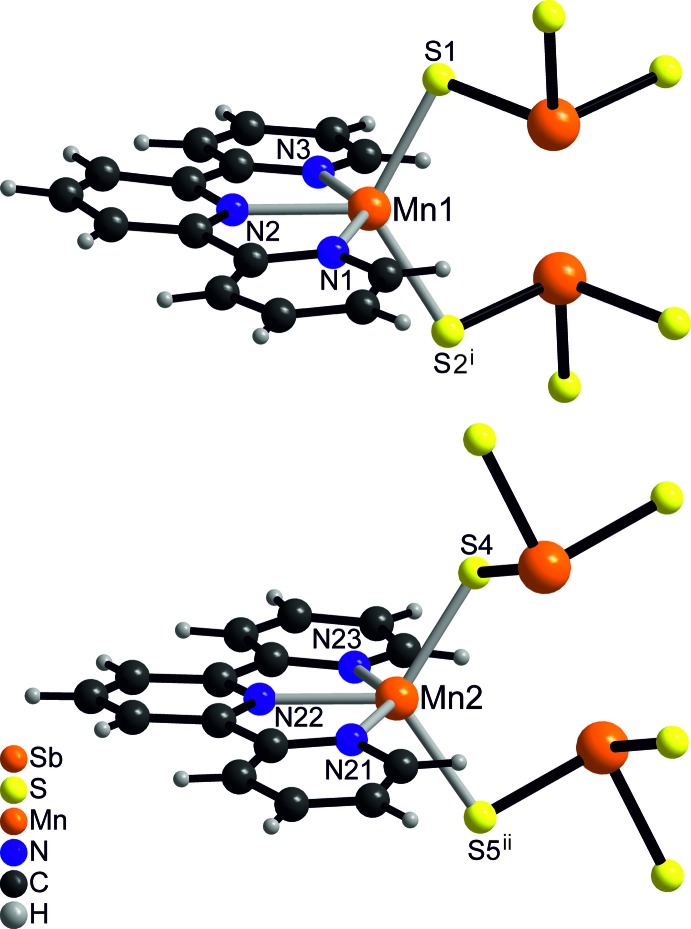
View of the Mn coordination sphere for Mn1 (top) and Mn2 (bottom). Symmetry codes used to generate symmetry-equivalent atoms: (i) −*x* + 1, −*y* + 1, −*z* + 1; (ii) −*x* + 1, −*y* + 1, −*z* + 2].

**Figure 3:**
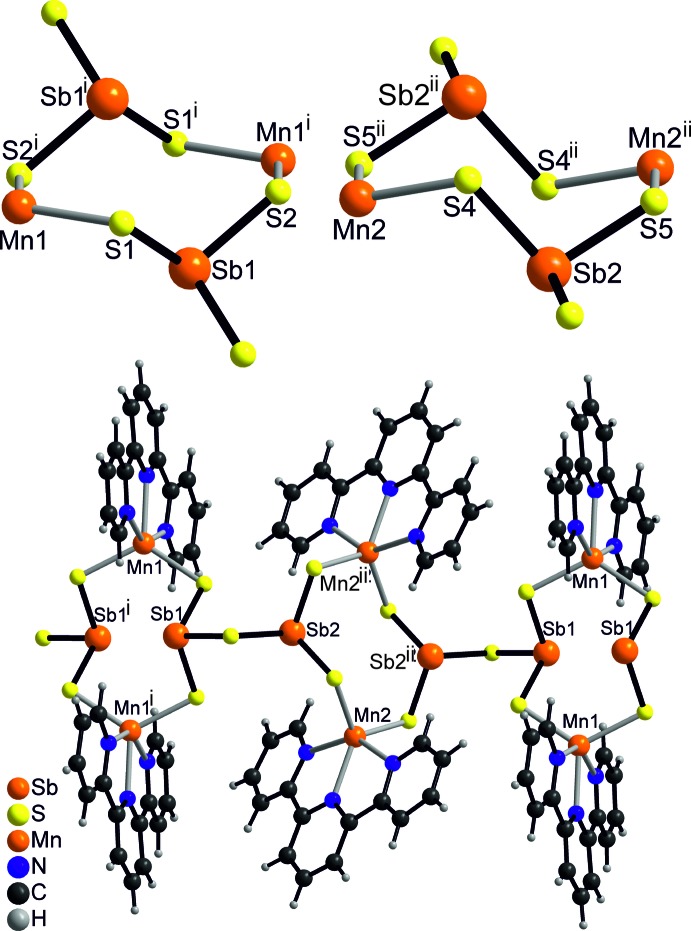
View of the eight-membered Mn_2_Sb_2_S_4_ rings for Mn1 (top: left) and Mn2 (top: right) as well as of the Mn_2_Sb_2_S_5_ chains (bottom). Symmetry codes used to generate symmetry-equivalent atoms: (i) −*x* + 1, −*y* + 1, −*z* + 1; (ii) −*x* + 1, −*y* + 1, −*z* + 2].

The asymmetric unit of the title compound consists of one [Sb_2_S_5_]^4−^ anion, two [Mn(terpyridine)]^2+^ cations and four solvent water mol­ecules in general positions (Fig. 1 ▸). Each Mn^II^ ion is fivefold coordinated by the three N atoms of the terpyridine ligand and two S atoms of two [Sb_2_S_5_]^4−^ anions that are related by symmetry (Fig. 2 ▸). The Mn—N and Mn—S distances are very similar for both independent Mn^II^ ions and correspond to literature values (Table 1 ▸). The Mn coordination environment is highly distorted with the three N atoms of the neutral terpyridine ligand and the Mn^II^ ion in the same plane and the two S atoms above and below this plane, leading to an irregular coordination (Fig. 1 ▸ and Table 1 ▸). The [Sb_2_S_5_]^4−^ anion consists of two trigonal–pyramidal SbS_3_ units that are linked by common corners (Fig. 3 ▸: top). The Sb—S bond lengths to the bridging S atom S3 are significantly longer than that to the terminal S atoms (Table 1 ▸). Two such anions are linked into eight-membered Mn_2_Sb_2_S_4_ rings that are located on centers of inversion and show a chair-like conformation. Two crystallographically independent rings are present that either contain Mn1 or Mn2 and which show a significantly different conformation (Fig. 3 ▸: top and Table 1 ▸). The Mn^II^ ions are each linked by two [Mn(terpyridine)]^2+^ cations into chains in the *c*-axis direction (Fig. 3 ▸: bottom). It is noted that this topology of the MnSbS network is completely different from that observed in all other Mn_2_Sb_2_S_5_ compounds with N-donor coligands (see above and *Database survey*).

## Supra­molecular features   

**Figure 4:**
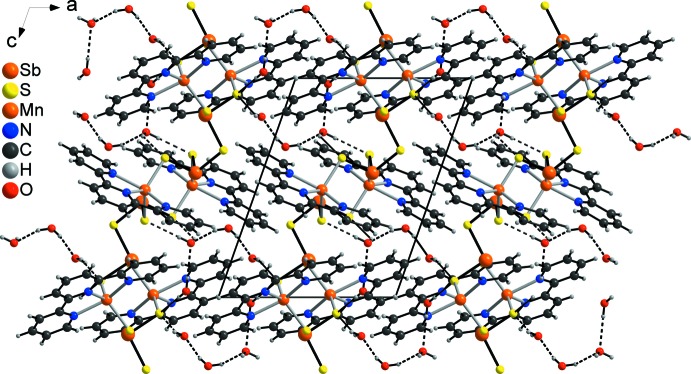
Crystal packing of the title compound viewed along the *b* axis with inter­molecular O—H⋯O and O—H⋯S hydrogen bonds shown as dashed lines.

In the crystal of the title compound, the MnSbS chains are linked to the solvent water mol­ecules by strong inter­molecular O—H⋯S hydrogen bonds (Fig. 4 ▸ and Table 2 ▸). The water mol­ecules of neighbouring chains are inter­linked by additional water mol­ecules *via* strong inter­molecular O—H⋯O hydrogen bonds into a three-dimensional network (Fig. 4 ▸ and Table 2 ▸). There are additional C—H⋯S and C—H⋯O inter­actions, but most of the C—H⋯S and C—H⋯O angles are far from linearity and thus, they should represent relatively weak inter­actions (Table 2 ▸).

## Database survey   

There are a number of other manganese thio­anti­monates with the general formula Mn_2_ *L*Sb_2_S_5_ or Mn_2_ *L*_2_Sb_2_S_5_ (*L* = amine ligand) reported in the literature that contain neutral Mn_2_Sb_2_S_5_ units and additional N-donor coligands. This includes Mn_2_(methyl­amino)_2_Sb_2_S_5_ and Mn_2_(1,3-di­amino­propane)Sb_2_S_5_ as well as Mn_2_(ethyl­enedi­amine)_2_Sb_2_S_5_ Mn_2_(ethyl­amino)_2_Sb_2_S_5_, with the latter showing a reversible phase transition (Bensch & Schur, 1996 ▸; Schur & Bensch, 2002 ▸; Schur *et al.*, 2001 ▸). This also includes Mn_2_(1,3-di­amino­pentene)Sb_2_S_5_ and two further compounds with di­ethyl­enetri­amine and *N*-methyl-1,3-di­amino­propane as ligands (Puls, *et al.*, 2006 ▸; Engelke *et al.*, 2004 ▸). Amongst these Mn compounds, there are some others with different transition metal cations such as, for example, Cu^II^ or Co^II^ (Spetzler *et al.*, 2005 ▸; Stähler & Bensch, 2001 ▸).

For reviews of chalcogenido thio­metallates including thio­anti­monates, see: Sheldrick & Wachhold (1998 ▸); Dehnen & Melullis (2007 ▸); Zhou *et al.* (2009 ▸); Seidlhofer *et al.* (2010 ▸); Wang *et al.* (2016 ▸); Zhou (2016 ▸); Zhu & Dai (2017 ▸).

## Synthesis and crystallization   

**General:** Na_3_SbS_3_ was prepared by the reaction of anhydrous Na_2_S (ABCR, 95%), Sb (99.5%, Sigma Aldrich) and sulfur (99%, ABCR) in a molar ratio of 3:2:3 at 870 K in a silica glass ampoule according to a literature procedure (Pompe & Pfitzner, 2013 ▸). The pale-yellow compound is sensitive to air and moisture and must be stored under a nitro­gen atmosphere.

Mn(terpy)_2_(ClO_4_)_2_] was prepared according to the literature (Rao *et al.*, 1976 ▸). 0.5 mmol of Mn(ClO_4_)_2_·6H_2_O (ABCR 99%) was dissolved in 25 mL of dry ethanol. Another solution containing 1.2 mmol of 2,2′;6′,2′′-terpyridine (ABCR 97%) was added to the the first solution. Upon mixing, a yellow solid precipitated that was filtered off and recrystallized from dry ethanol.

**Synthesis:**

Single crystals of the title compound were obtained by adding 2 mL of H_2_O in a glass tube to a mixture of 72.0 mg (0.1 mmol) Mn(terpy)_2_(ClO_4_)_2_ and 57.4 mg (0.2 mmol) of Na_3_SbS_3_. The slurry was heated to 413 K for 2 h. After cooling to room temperature, small red needles with a yield of 10% were obtained together with a very small amount of an unknown crystalline phase and of a colourless solid that is amorphous against X-rays.

**Experimental methods:**

The XRPD measurements were performed by using a Stoe Transmission Powder Diffraction System (STADI P) with Cu *K*α radiation that was equipped with a linear, position-sensitive MYTHEN detector from Stoe & Cie.

## Refinement   

Crystal data, data collection and structure refinement details are summarized in Table 3 ▸. Hydrogen atoms were positioned with idealized geometry and were refined with *U*_iso_(H) = 1.2*U*_eq_(C) using a riding model. Some of the water H atoms were located in a difference-Fourier map; their bond lengths were set to ideal values and finally they were refined isotropically with *U*_iso_(H) = 1.5*U*_eq_(O). The water H atoms that could not be located in a difference-Fourier map were included in idealized calculated positions that gave the most sensible geometry as donors for hydrogen bonds.

The crystal studied was twinned by non-merohedry around a pseudo twofold rotation axis, with a matrix close to 0 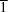 0 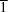 0 0 0 0 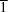 but refinement in *SHELXL* (Sheldrick, 2015 ▸) assuming this kind of twinning lead to only very poor reliability factors. Therefore, both individual domains were indexed separately and the overlapping reflections were removed. In this case, relatively good reliability factors were observed but the completeness was only 68.6%. Thus, the data were integrated neglecting the twinning, corrected for absorption and merged. Afterwards the twin law was determined and the data were transformed into HKLF-5 format (Sheldrick, 2015 ▸), leading to full completeness and acceptable reliability factors.

## Supplementary Material

Crystal structure: contains datablock(s) I. DOI: 10.1107/S2056989019016268/lh5938sup1.cif

Structure factors: contains datablock(s) I. DOI: 10.1107/S2056989019016268/lh5938Isup2.hkl

Fig. S1. Experimental and simulated X-ray powder pattern for the title compound. DOI: 10.1107/S2056989019016268/lh5938sup3.jpg

CCDC reference: 1969700

Additional supporting information:  crystallographic information; 3D view; checkCIF report

## Figures and Tables

**Table 1:** Selected geometric parameters (Å, °)

Sb1—S2	2.391 (2)	S2—Mn1^i^	2.414 (3)
Sb1—S1	2.404 (2)	S4—Mn2	2.411 (3)
Sb1—S3	2.445 (2)	S5—Mn2^ii^	2.405 (3)
Sb2—S5	2.396 (2)	Mn1—N2	2.228 (7)
Sb2—S4	2.402 (2)	Mn1—N3	2.258 (7)
Sb2—S3	2.467 (3)	Mn1—N1	2.285 (8)
S1—Mn1	2.419 (3)		
			
S2—Sb1—S1	100.84 (9)	N2—Mn1—N3	71.6 (3)
S2—Sb1—S3	97.77 (8)	N2—Mn1—N1	71.9 (3)
S1—Sb1—S3	98.41 (8)	N3—Mn1—N1	143.5 (3)
S5—Sb2—S4	99.08 (9)	N2—Mn1—S2^i^	118.1 (2)
S5—Sb2—S3	93.00 (8)	N3—Mn1—S2^i^	93.7 (2)
S4—Sb2—S3	96.64 (9)	N1—Mn1—S2^i^	105.6 (2)
Sb1—S1—Mn1	102.22 (9)	N2—Mn1—S1	122.2 (2)
Sb1—S2—Mn1^i^	100.17 (10)	N3—Mn1—S1	103.9 (2)
Sb1—S3—Sb2	100.47 (9)	N1—Mn1—S1	93.1 (2)
Sb2—S4—Mn2	109.95 (10)	S2^i^—Mn1—S1	119.71 (10)
Sb2—S5—Mn2^ii^	98.38 (10)		

Symmetry codes: (i) 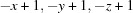; (ii) 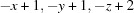.

**Table 2:** Hydrogen-bond geometry (Å, °)

*D*—H⋯*A*	*D*—H	H⋯*A*	*D*⋯*A*	*D*—H⋯*A*
O1—H1*A*⋯S1	0.84	2.63	3.239 (10)	131
O1—H1*B*⋯S2	0.84	2.44	3.283 (11)	180
O2—H2*A*⋯O1	0.84	2.20	2.897 (19)	140
O2—H2*B*⋯O3	0.84	2.04	2.87 (2)	170
O3—H3*A*⋯S4^iii^	0.84	2.71	3.490 (14)	154
O3—H3*B*⋯S5^iii^	0.84	2.82	3.427 (14)	131
O4—H4*A*⋯O1	0.84	2.23	3.07 (2)	180
O4—H4*B*⋯S4^ii^	0.84	2.33	3.165 (17)	180
C4—H4⋯S3^iv^	0.95	2.81	3.747 (12)	170
C7—H7⋯S3^iv^	0.95	2.93	3.831 (12)	158
C9—H9⋯S3^v^	0.95	2.97	3.690 (10)	134
C9—H9⋯S5^v^	0.95	3.02	3.706 (11)	130
C12—H12⋯S1^v^	0.95	2.86	3.657 (10)	142
C21—H21⋯O4	0.95	2.34	3.15 (2)	143
C24—H24⋯S5^vi^	0.95	2.83	3.652 (15)	145
C29—H29⋯O4^vii^	0.95	2.12	2.98 (3)	150
C32—H32⋯S4^viii^	0.95	2.97	3.599 (13)	125

Symmetry codes: (ii) 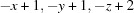; (iii) 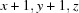; (iv) 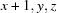; (v) 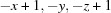; (vi) 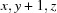; (vii) 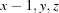; (viii) 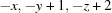.

**Table 3:** Experimental details

Crystal data	
Chemical formula	[Mn_2_Sb_2_S_5_(C_15_H_11_N_3_)_2_]·4H_2_O
*M*_r_	1052.28
Crystal system, space group	Triclinic, *P* 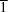
Temperature (K)	200
*a*, *b*, *c* (Å)	11.9227 (5), 12.1592 (6), 14.9217 (7)
α, β, γ (°)	104.293 (3), 101.701 (3), 112.585 (3)
*V* (Å^3^)	1825.27 (15)
*Z*	2
Radiation type	Mo *K*α
μ (mm^−1^)	2.47
Crystal size (mm)	0.13 × 0.08 × 0.06

Data collection	
Diffractometer	Stoe *IPDS2*
Absorption correction	Numerical (*X-RED* and *X-SHAPE*; Stoe & Cie, 2008 ▸)
*T*_min_, *T*_max_	0.624, 0.748
No. of measured, independent and observed [*I* > 2σ(*I*)] reflections	7084, 7084, 5834
(sin θ/λ)_max_ (Å^−1^)	0.621

Refinement	
*R*[*F* ^2^ > 2σ(*F* ^2^)], *wR*(*F* ^2^), *S*	0.054, 0.182, 1.07
No. of reflections	7084
No. of parameters	444
H-atom treatment	H-atom parameters constrained
Δρ_max_, Δρ_min_ (e Å^−3^)	1.08, −0.98

Computer programs: *X-AREA* (Stoe & Cie, 2008 ▸), *SHELXS97* (Sheldrick, 2008 ▸), *SHELXL2018* (Sheldrick, 2015 ▸), *DIAMOND* (Brandenburg, 1999 ▸), *publCIF* (Westrip, 2010 ▸).

## supplementary crystallographic information

### Crystal data

**Table:**

[Mn_2_Sb_2_S_5_(C_15_H_11_N_3_)_2_]·4H_2_O	*Z* = 2
*M**_r_* = 1052.28	*F*(000) = 1032
Triclinic, *P*1	*D*_x_ = 1.915 Mg m^−^^3^
*a* = 11.9227 (5) Å	Mo *K*α radiation, λ = 0.71073 Å
*b* = 12.1592 (6) Å	Cell parameters from 12925 reflections
*c* = 14.9217 (7) Å	θ = 1.5–26.2°
α = 104.293 (3)°	µ = 2.47 mm^−^^1^
β = 101.701 (3)°	*T* = 200 K
γ = 112.585 (3)°	Block, red
*V* = 1825.27 (15) Å^3^	0.13 × 0.08 × 0.06 mm

### Data collection

**Table:**

Stoe IPDS-2 diffractometer	5834 reflections with *I* > 2σ(*I*)
ω scans	θ_max_ = 26.2°, θ_min_ = 1.5°
Absorption correction: numerical (X-Red and X-Shape; Stoe & Cie, 2008)	*h* = −14→14
*T*_min_ = 0.624, *T*_max_ = 0.748	*k* = −14→14
7084 measured reflections	*l* = −9→18
7084 independent reflections	

### Refinement

**Table:**

Refinement on *F*^2^	Hydrogen site location: mixed
Least-squares matrix: full	H-atom parameters constrained
*R*[*F*^2^ > 2σ(*F*^2^)] = 0.054	*w* = 1/[σ^2^(*F*_o_^2^) + (0.1094*P*)^2^ + 7.9201*P*] where *P* = (*F*_o_^2^ + 2*F*_c_^2^)/3
*wR*(*F*^2^) = 0.182	(Δ/σ)_max_ < 0.001
*S* = 1.07	Δρ_max_ = 1.08 e Å^−^^3^
7084 reflections	Δρ_min_ = −0.98 e Å^−^^3^
444 parameters	Extinction correction: SHELXL2018 (Sheldrick, 2015), Fc^*^=kFc[1+0.001xFc^2^λ^3^/sin(2θ)]^-1/4^
0 restraints	Extinction coefficient: 0.0156 (14)

### Special details

**Table:**

Geometry. All esds (except the esd in the dihedral angle between two l.s. planes) are estimated using the full covariance matrix. The cell esds are taken into account individually in the estimation of esds in distances, angles and torsion angles; correlations between esds in cell parameters are only used when they are defined by crystal symmetry. An approximate (isotropic) treatment of cell esds is used for estimating esds involving l.s. planes.
Refinement. Refined as a two-component twin.

### Fractional atomic coordinates and isotropic or equivalent isotropic displacement parameters (Å^2^)

**Table:**

	*x*	*y*	*z*	*U*_iso_*/*U*_eq_	
Sb1	0.37778 (6)	0.39853 (6)	0.56716 (4)	0.0362 (2)	
Sb2	0.43666 (6)	0.42290 (5)	0.83131 (4)	0.0347 (2)	
S1	0.5789 (2)	0.3987 (2)	0.63453 (17)	0.0391 (5)	
S2	0.4300 (3)	0.6161 (2)	0.64977 (18)	0.0409 (5)	
S3	0.2644 (2)	0.3063 (2)	0.67019 (19)	0.0410 (5)	
S4	0.3019 (2)	0.4485 (2)	0.9261 (2)	0.0460 (6)	
S5	0.4355 (2)	0.2315 (2)	0.84793 (19)	0.0428 (5)	
Mn1	0.62903 (13)	0.32502 (12)	0.48902 (10)	0.0347 (3)	
N1	0.8453 (8)	0.4535 (7)	0.5649 (6)	0.0404 (17)	
N2	0.7276 (8)	0.2017 (7)	0.4772 (6)	0.0368 (16)	
N3	0.4774 (7)	0.1195 (7)	0.4132 (6)	0.0364 (16)	
C1	0.8982 (10)	0.5779 (9)	0.6095 (7)	0.043 (2)	
H1	0.843905	0.618064	0.606305	0.052*	
C2	1.0295 (11)	0.6544 (10)	0.6615 (9)	0.053 (3)	
H2	1.063976	0.744043	0.692727	0.064*	
C3	1.1084 (12)	0.5942 (11)	0.6657 (10)	0.057 (3)	
H3	1.198416	0.642938	0.699585	0.068*	
C4	1.0541 (10)	0.4624 (11)	0.6200 (9)	0.052 (3)	
H4	1.105988	0.419606	0.623773	0.063*	
C5	0.9220 (10)	0.3942 (9)	0.5685 (8)	0.041 (2)	
C6	0.8559 (9)	0.2540 (9)	0.5134 (8)	0.041 (2)	
C7	0.9208 (11)	0.1819 (10)	0.4992 (10)	0.054 (3)	
H7	1.012020	0.220266	0.526538	0.065*	
C8	0.8517 (12)	0.0550 (11)	0.4452 (9)	0.057 (3)	
H8	0.895377	0.005087	0.432484	0.069*	
C9	0.7186 (11)	−0.0023 (10)	0.4085 (8)	0.049 (2)	
H9	0.669897	−0.091173	0.371658	0.059*	
C10	0.6575 (9)	0.0760 (8)	0.4277 (7)	0.0361 (19)	
C11	0.5173 (10)	0.0297 (8)	0.3953 (6)	0.0357 (18)	
C12	0.4295 (11)	−0.1001 (9)	0.3504 (7)	0.044 (2)	
H12	0.459932	−0.162278	0.340345	0.052*	
C13	0.3003 (11)	−0.1376 (9)	0.3211 (7)	0.045 (2)	
H13	0.240224	−0.225324	0.289009	0.054*	
C14	0.2583 (11)	−0.0453 (10)	0.3390 (8)	0.050 (2)	
H14	0.169008	−0.068183	0.320257	0.060*	
C15	0.3504 (10)	0.0818 (9)	0.3851 (7)	0.043 (2)	
H15	0.321825	0.145279	0.397316	0.052*	
Mn2	0.37319 (16)	0.67226 (14)	1.01113 (11)	0.0413 (4)	
N21	0.4598 (13)	0.7905 (9)	0.9250 (7)	0.062 (3)	
N22	0.2544 (11)	0.7693 (9)	0.9747 (6)	0.054 (2)	
N23	0.2195 (9)	0.6283 (8)	1.0844 (6)	0.0436 (18)	
C21	0.5691 (16)	0.8057 (11)	0.9108 (9)	0.069 (4)	
H21	0.608757	0.757593	0.931466	0.083*	
C22	0.630 (2)	0.8900 (13)	0.8665 (10)	0.090 (6)	
H22	0.709957	0.900042	0.858841	0.109*	
C23	0.573 (3)	0.9559 (12)	0.8351 (10)	0.105 (8)	
H23	0.611734	1.012190	0.803711	0.126*	
C24	0.460 (2)	0.9413 (13)	0.8487 (9)	0.091 (7)	
H24	0.417808	0.986953	0.826740	0.110*	
C25	0.4057 (19)	0.8587 (11)	0.8951 (8)	0.074 (5)	
C26	0.2881 (17)	0.8444 (11)	0.9202 (8)	0.070 (4)	
C27	0.209 (3)	0.9006 (17)	0.8945 (10)	0.113 (9)	
H27	0.228365	0.950793	0.854799	0.135*	
C28	0.105 (3)	0.8844 (19)	0.9258 (12)	0.109 (8)	
H28	0.054054	0.924278	0.908344	0.131*	
C29	0.0757 (17)	0.8124 (17)	0.9811 (11)	0.086 (5)	
H29	0.003705	0.800505	1.002708	0.103*	
C30	0.1548 (13)	0.7546 (11)	1.0064 (9)	0.057 (3)	
C31	0.1317 (10)	0.6736 (11)	1.0656 (8)	0.051 (3)	
C32	0.0314 (12)	0.6440 (12)	1.0990 (10)	0.064 (3)	
H32	−0.028291	0.676671	1.085717	0.076*	
C33	0.0169 (11)	0.5651 (14)	1.1530 (10)	0.070 (4)	
H33	−0.054269	0.542127	1.176270	0.085*	
C34	0.1030 (12)	0.5205 (12)	1.1730 (9)	0.058 (3)	
H34	0.094179	0.466582	1.210361	0.069*	
C35	0.2051 (11)	0.5563 (12)	1.1371 (8)	0.051 (2)	
H35	0.267249	0.526746	1.151685	0.061*	
O1	0.7367 (10)	0.7040 (9)	0.7526 (8)	0.087 (3)	
H1A	0.747033	0.638528	0.734270	0.131*	
H1B	0.658273	0.681508	0.726219	0.131*	
O2	0.9205 (14)	0.8717 (13)	0.6860 (10)	0.102 (4)	
H2A	0.848835	0.842934	0.694566	0.153*	
H2B	0.981845	0.944124	0.720865	0.153*	
O3	1.1152 (14)	1.1177 (13)	0.8230 (13)	0.124 (5)	
H3A	1.150095	1.194473	0.827743	0.186*	
H3B	1.178715	1.104813	0.842072	0.186*	
O4	0.8058 (16)	0.7512 (19)	0.9728 (15)	0.146 (7)	
H4A	0.787124	0.738460	0.912575	0.219*	
H4B	0.777494	0.698331	0.999835	0.219*	

### Atomic displacement parameters (Å^2^)

**Table:**

	*U*^11^	*U*^22^	*U*^33^	*U*^12^	*U*^13^	*U*^23^
Sb1	0.0422 (4)	0.0341 (3)	0.0340 (3)	0.0206 (3)	0.0104 (3)	0.0116 (2)
Sb2	0.0375 (3)	0.0307 (3)	0.0394 (3)	0.0173 (2)	0.0150 (3)	0.0136 (2)
S1	0.0417 (12)	0.0417 (12)	0.0393 (12)	0.0262 (10)	0.0118 (10)	0.0127 (10)
S2	0.0495 (13)	0.0332 (11)	0.0444 (12)	0.0229 (10)	0.0145 (10)	0.0154 (9)
S3	0.0427 (12)	0.0335 (11)	0.0476 (13)	0.0167 (10)	0.0142 (10)	0.0179 (10)
S4	0.0438 (13)	0.0386 (12)	0.0571 (15)	0.0202 (10)	0.0247 (12)	0.0106 (11)
S5	0.0453 (13)	0.0337 (11)	0.0514 (13)	0.0215 (10)	0.0121 (11)	0.0160 (10)
Mn1	0.0358 (7)	0.0303 (7)	0.0394 (7)	0.0173 (6)	0.0120 (6)	0.0116 (6)
N1	0.034 (4)	0.033 (4)	0.048 (4)	0.014 (3)	0.010 (3)	0.012 (3)
N2	0.043 (4)	0.034 (4)	0.046 (4)	0.026 (3)	0.020 (4)	0.016 (3)
N3	0.035 (4)	0.028 (4)	0.039 (4)	0.012 (3)	0.009 (3)	0.008 (3)
C1	0.038 (5)	0.035 (5)	0.047 (5)	0.015 (4)	0.010 (4)	0.005 (4)
C2	0.043 (6)	0.038 (5)	0.062 (7)	0.008 (4)	0.014 (5)	0.011 (5)
C3	0.043 (6)	0.045 (6)	0.068 (7)	0.016 (5)	0.013 (5)	0.007 (5)
C4	0.035 (5)	0.048 (6)	0.064 (7)	0.017 (5)	0.011 (5)	0.013 (5)
C5	0.040 (5)	0.036 (5)	0.054 (6)	0.023 (4)	0.018 (4)	0.014 (4)
C6	0.036 (5)	0.034 (5)	0.054 (6)	0.020 (4)	0.014 (4)	0.009 (4)
C7	0.038 (5)	0.038 (5)	0.080 (8)	0.021 (4)	0.008 (5)	0.012 (5)
C8	0.055 (7)	0.039 (5)	0.066 (7)	0.028 (5)	0.006 (6)	0.003 (5)
C9	0.058 (6)	0.032 (5)	0.058 (6)	0.030 (5)	0.013 (5)	0.006 (4)
C10	0.040 (5)	0.026 (4)	0.044 (5)	0.020 (4)	0.011 (4)	0.009 (4)
C11	0.051 (5)	0.027 (4)	0.032 (4)	0.020 (4)	0.013 (4)	0.011 (3)
C12	0.061 (6)	0.032 (4)	0.037 (5)	0.020 (4)	0.013 (4)	0.014 (4)
C13	0.053 (6)	0.024 (4)	0.043 (5)	0.010 (4)	0.012 (4)	0.005 (4)
C14	0.040 (5)	0.045 (6)	0.052 (6)	0.014 (4)	0.010 (5)	0.008 (5)
C15	0.052 (6)	0.035 (5)	0.042 (5)	0.022 (4)	0.018 (4)	0.009 (4)
Mn2	0.0557 (9)	0.0386 (8)	0.0376 (7)	0.0287 (7)	0.0152 (7)	0.0150 (6)
N21	0.106 (9)	0.040 (5)	0.042 (5)	0.031 (5)	0.032 (5)	0.020 (4)
N22	0.070 (6)	0.048 (5)	0.038 (4)	0.038 (5)	−0.004 (4)	0.005 (4)
N23	0.050 (5)	0.046 (4)	0.043 (4)	0.032 (4)	0.014 (4)	0.013 (4)
C21	0.110 (11)	0.034 (5)	0.048 (6)	0.017 (6)	0.039 (7)	0.007 (5)
C22	0.142 (16)	0.051 (7)	0.051 (7)	0.011 (9)	0.050 (9)	0.015 (6)
C23	0.21 (2)	0.034 (6)	0.038 (7)	0.022 (10)	0.041 (11)	0.014 (5)
C24	0.19 (2)	0.040 (6)	0.034 (6)	0.042 (9)	0.030 (9)	0.012 (5)
C25	0.145 (14)	0.036 (6)	0.029 (5)	0.038 (7)	0.017 (7)	0.010 (4)
C26	0.127 (13)	0.045 (6)	0.034 (5)	0.049 (7)	0.001 (7)	0.009 (5)
C27	0.23 (3)	0.085 (11)	0.036 (6)	0.113 (15)	0.001 (10)	0.011 (7)
C28	0.19 (2)	0.106 (13)	0.061 (9)	0.122 (16)	0.006 (11)	0.017 (9)
C29	0.091 (11)	0.097 (11)	0.070 (9)	0.073 (10)	−0.006 (8)	0.007 (8)
C30	0.065 (7)	0.045 (6)	0.052 (6)	0.038 (6)	−0.006 (5)	−0.001 (5)
C31	0.040 (5)	0.055 (6)	0.048 (6)	0.032 (5)	0.001 (4)	−0.002 (5)
C32	0.040 (6)	0.052 (6)	0.077 (8)	0.020 (5)	0.001 (6)	0.007 (6)
C33	0.032 (6)	0.073 (8)	0.067 (8)	0.012 (5)	0.008 (5)	−0.013 (7)
C34	0.057 (7)	0.059 (7)	0.049 (6)	0.022 (6)	0.021 (5)	0.012 (5)
C35	0.044 (6)	0.066 (7)	0.043 (5)	0.027 (5)	0.015 (4)	0.018 (5)
O1	0.068 (6)	0.054 (5)	0.090 (7)	0.022 (5)	−0.012 (5)	−0.011 (5)
O2	0.109 (10)	0.092 (8)	0.107 (9)	0.055 (8)	0.033 (8)	0.026 (7)
O3	0.096 (10)	0.078 (8)	0.200 (16)	0.028 (7)	0.071 (10)	0.056 (9)
O4	0.109 (12)	0.157 (16)	0.190 (18)	0.066 (11)	0.034 (12)	0.096 (14)

### Geometric parameters (Å, º)

**Table:**

Sb1—S2	2.391 (2)	C15—H15	0.9500
Sb1—S1	2.404 (2)	Mn2—N22	2.233 (9)
Sb1—S3	2.445 (2)	Mn2—N21	2.257 (9)
Sb2—S5	2.396 (2)	Mn2—N23	2.278 (9)
Sb2—S4	2.402 (2)	N21—C21	1.31 (2)
Sb2—S3	2.467 (3)	N21—C25	1.334 (18)
S1—Mn1	2.419 (3)	N22—C30	1.331 (17)
S2—Mn1^i^	2.414 (3)	N22—C26	1.368 (17)
S4—Mn2	2.411 (3)	N23—C35	1.301 (14)
S5—Mn2^ii^	2.405 (3)	N23—C31	1.371 (13)
Mn1—N2	2.228 (7)	C21—C22	1.405 (17)
Mn1—N3	2.258 (7)	C21—H21	0.9500
Mn1—N1	2.285 (8)	C22—C23	1.34 (3)
N1—C1	1.315 (13)	C22—H22	0.9500
N1—C5	1.366 (13)	C23—C24	1.36 (3)
N2—C6	1.336 (13)	C23—H23	0.9500
N2—C10	1.339 (12)	C24—C25	1.39 (2)
N3—C15	1.339 (13)	C24—H24	0.9500
N3—C11	1.341 (12)	C25—C26	1.48 (2)
C1—C2	1.397 (15)	C26—C27	1.40 (2)
C1—H1	0.9500	C27—C28	1.37 (3)
C2—C3	1.396 (17)	C27—H27	0.9500
C2—H2	0.9500	C28—C29	1.34 (3)
C3—C4	1.393 (16)	C28—H28	0.9500
C3—H3	0.9500	C29—C30	1.422 (17)
C4—C5	1.397 (15)	C29—H29	0.9500
C4—H4	0.9500	C30—C31	1.465 (18)
C5—C6	1.488 (13)	C31—C32	1.344 (18)
C6—C7	1.382 (14)	C32—C33	1.38 (2)
C7—C8	1.360 (15)	C32—H32	0.9500
C7—H7	0.9500	C33—C34	1.35 (2)
C8—C9	1.383 (17)	C33—H33	0.9500
C8—H8	0.9500	C34—C35	1.390 (16)
C9—C10	1.415 (13)	C34—H34	0.9500
C9—H9	0.9500	C35—H35	0.9500
C10—C11	1.469 (14)	O1—H1A	0.8400
C11—C12	1.400 (13)	O1—H1B	0.8401
C12—C13	1.364 (16)	O2—H2A	0.8400
C12—H12	0.9500	O2—H2B	0.8400
C13—C14	1.385 (16)	O3—H3A	0.8400
C13—H13	0.9500	O3—H3B	0.8400
C14—C15	1.390 (14)	O4—H4A	0.8399
C14—H14	0.9500	O4—H4B	0.8401
			
S2—Sb1—S1	100.84 (9)	N3—C15—C14	123.4 (10)
S2—Sb1—S3	97.77 (8)	N3—C15—H15	118.3
S1—Sb1—S3	98.41 (8)	C14—C15—H15	118.3
S5—Sb2—S4	99.08 (9)	N22—Mn2—N21	72.0 (4)
S5—Sb2—S3	93.00 (8)	N22—Mn2—N23	71.3 (4)
S4—Sb2—S3	96.64 (9)	N21—Mn2—N23	143.0 (4)
Sb1—S1—Mn1	102.22 (9)	N22—Mn2—S5^ii^	123.9 (3)
Sb1—S2—Mn1^i^	100.17 (10)	N21—Mn2—S5^ii^	96.5 (3)
Sb1—S3—Sb2	100.47 (9)	N23—Mn2—S5^ii^	100.3 (2)
Sb2—S4—Mn2	109.95 (10)	N22—Mn2—S4	122.1 (3)
Sb2—S5—Mn2^ii^	98.38 (10)	N21—Mn2—S4	111.3 (3)
N2—Mn1—N3	71.6 (3)	N23—Mn2—S4	91.9 (2)
N2—Mn1—N1	71.9 (3)	S5^ii^—Mn2—S4	113.34 (10)
N3—Mn1—N1	143.5 (3)	C21—N21—C25	117.5 (12)
N2—Mn1—S2^i^	118.1 (2)	C21—N21—Mn2	122.8 (9)
N3—Mn1—S2^i^	93.7 (2)	C25—N21—Mn2	119.3 (11)
N1—Mn1—S2^i^	105.6 (2)	C30—N22—C26	121.8 (11)
N2—Mn1—S1	122.2 (2)	C30—N22—Mn2	120.2 (8)
N3—Mn1—S1	103.9 (2)	C26—N22—Mn2	118.0 (10)
N1—Mn1—S1	93.1 (2)	C35—N23—C31	118.4 (10)
S2^i^—Mn1—S1	119.71 (10)	C35—N23—Mn2	124.1 (7)
C1—N1—C5	118.9 (9)	C31—N23—Mn2	117.3 (7)
C1—N1—Mn1	124.3 (7)	N21—C21—C22	122.8 (16)
C5—N1—Mn1	116.7 (6)	N21—C21—H21	118.6
C6—N2—C10	120.5 (8)	C22—C21—H21	118.6
C6—N2—Mn1	120.2 (6)	C23—C22—C21	119 (2)
C10—N2—Mn1	119.2 (6)	C23—C22—H22	120.5
C15—N3—C11	118.1 (8)	C21—C22—H22	120.5
C15—N3—Mn1	124.1 (6)	C22—C23—C24	119.1 (14)
C11—N3—Mn1	117.8 (6)	C22—C23—H23	120.5
N1—C1—C2	123.8 (10)	C24—C23—H23	120.5
N1—C1—H1	118.1	C23—C24—C25	119.3 (17)
C2—C1—H1	118.1	C23—C24—H24	120.4
C3—C2—C1	117.6 (10)	C25—C24—H24	120.4
C3—C2—H2	121.2	N21—C25—C24	122.3 (18)
C1—C2—H2	121.2	N21—C25—C26	114.6 (11)
C4—C3—C2	119.6 (11)	C24—C25—C26	123.1 (15)
C4—C3—H3	120.2	N22—C26—C27	117.3 (18)
C2—C3—H3	120.2	N22—C26—C25	116.1 (11)
C3—C4—C5	118.7 (11)	C27—C26—C25	126.6 (15)
C3—C4—H4	120.7	C28—C27—C26	121.2 (17)
C5—C4—H4	120.7	C28—C27—H27	119.4
N1—C5—C4	121.5 (9)	C26—C27—H27	119.4
N1—C5—C6	115.7 (9)	C29—C28—C27	120.4 (15)
C4—C5—C6	122.9 (9)	C29—C28—H28	119.8
N2—C6—C7	121.7 (9)	C27—C28—H28	119.8
N2—C6—C5	115.1 (8)	C28—C29—C30	118.4 (18)
C7—C6—C5	123.2 (9)	C28—C29—H29	120.8
C8—C7—C6	118.7 (10)	C30—C29—H29	120.8
C8—C7—H7	120.7	N22—C30—C29	120.8 (14)
C6—C7—H7	120.7	N22—C30—C31	115.7 (9)
C7—C8—C9	120.9 (10)	C29—C30—C31	123.4 (14)
C7—C8—H8	119.5	C32—C31—N23	121.6 (12)
C9—C8—H8	119.5	C32—C31—C30	123.1 (11)
C8—C9—C10	117.7 (9)	N23—C31—C30	115.3 (10)
C8—C9—H9	121.2	C31—C32—C33	118.7 (12)
C10—C9—H9	121.2	C31—C32—H32	120.6
N2—C10—C9	120.4 (9)	C33—C32—H32	120.6
N2—C10—C11	115.1 (8)	C34—C33—C32	120.5 (12)
C9—C10—C11	124.4 (9)	C34—C33—H33	119.8
N3—C11—C12	121.3 (10)	C32—C33—H33	119.8
N3—C11—C10	115.9 (8)	C33—C34—C35	117.7 (13)
C12—C11—C10	122.8 (9)	C33—C34—H34	121.2
C13—C12—C11	120.2 (10)	C35—C34—H34	121.2
C13—C12—H12	119.9	N23—C35—C34	123.1 (12)
C11—C12—H12	119.9	N23—C35—H35	118.5
C12—C13—C14	118.8 (9)	C34—C35—H35	118.5
C12—C13—H13	120.6	H1A—O1—H1B	106.5
C14—C13—H13	120.6	H2A—O2—H2B	123.6
C13—C14—C15	118.2 (10)	H3A—O3—H3B	102.7
C13—C14—H14	120.9	H4A—O4—H4B	127.9
C15—C14—H14	120.9		

Symmetry codes: (i) −*x*+1, −*y*+1, −*z*+1; (ii) −*x*+1, −*y*+1, −*z*+2.

### Hydrogen-bond geometry (Å, º)

**Table:**

*D*—H···*A*	*D*—H	H···*A*	*D*···*A*	*D*—H···*A*
O1—H1*A*···S1	0.84	2.63	3.239 (10)	131
O1—H1*B*···S2	0.84	2.44	3.283 (11)	180
O2—H2*A*···O1	0.84	2.20	2.897 (19)	140
O2—H2*B*···O3	0.84	2.04	2.87 (2)	170
O3—H3*A*···S4^iii^	0.84	2.71	3.490 (14)	154
O3—H3*B*···S5^iii^	0.84	2.82	3.427 (14)	131
O4—H4*A*···O1	0.84	2.23	3.07 (2)	180
O4—H4*B*···S4^ii^	0.84	2.33	3.165 (17)	180
C4—H4···S3^iv^	0.95	2.81	3.747 (12)	170
C7—H7···S3^iv^	0.95	2.93	3.831 (12)	158
C9—H9···S3^v^	0.95	2.97	3.690 (10)	134
C9—H9···S5^v^	0.95	3.02	3.706 (11)	130
C12—H12···S1^v^	0.95	2.86	3.657 (10)	142
C21—H21···O4	0.95	2.34	3.15 (2)	143
C24—H24···S5^vi^	0.95	2.83	3.652 (15)	145
C29—H29···O4^vii^	0.95	2.12	2.98 (3)	150
C32—H32···S4^viii^	0.95	2.97	3.599 (13)	125

Symmetry codes: (ii) −*x*+1, −*y*+1, −*z*+2; (iii) *x*+1, *y*+1, *z*; (iv) *x*+1, *y*, *z*; (v) −*x*+1, −*y*, −*z*+1; (vi) *x*, *y*+1, *z*; (vii) *x*−1, *y*, *z*; (viii) −*x*, −*y*+1, −*z*+2.
