# Supplementary figures and images for: Synthesis and crystal structure of catena-poly[[bis[(2,2′;6′,2′′-terpyridine)­manganese(II)]-μ4-penta­thio­dianti­monato] tetra­hydrate] showing a 1D MnSbS network
Source: Acta Crystallogr E Crystallogr Commun. 2020 Jan 1;76(Pt 1):32–7. doi: 10.1107/S2056989019016268 (PMC6944084; doi:10.1107/S2056989019016268)

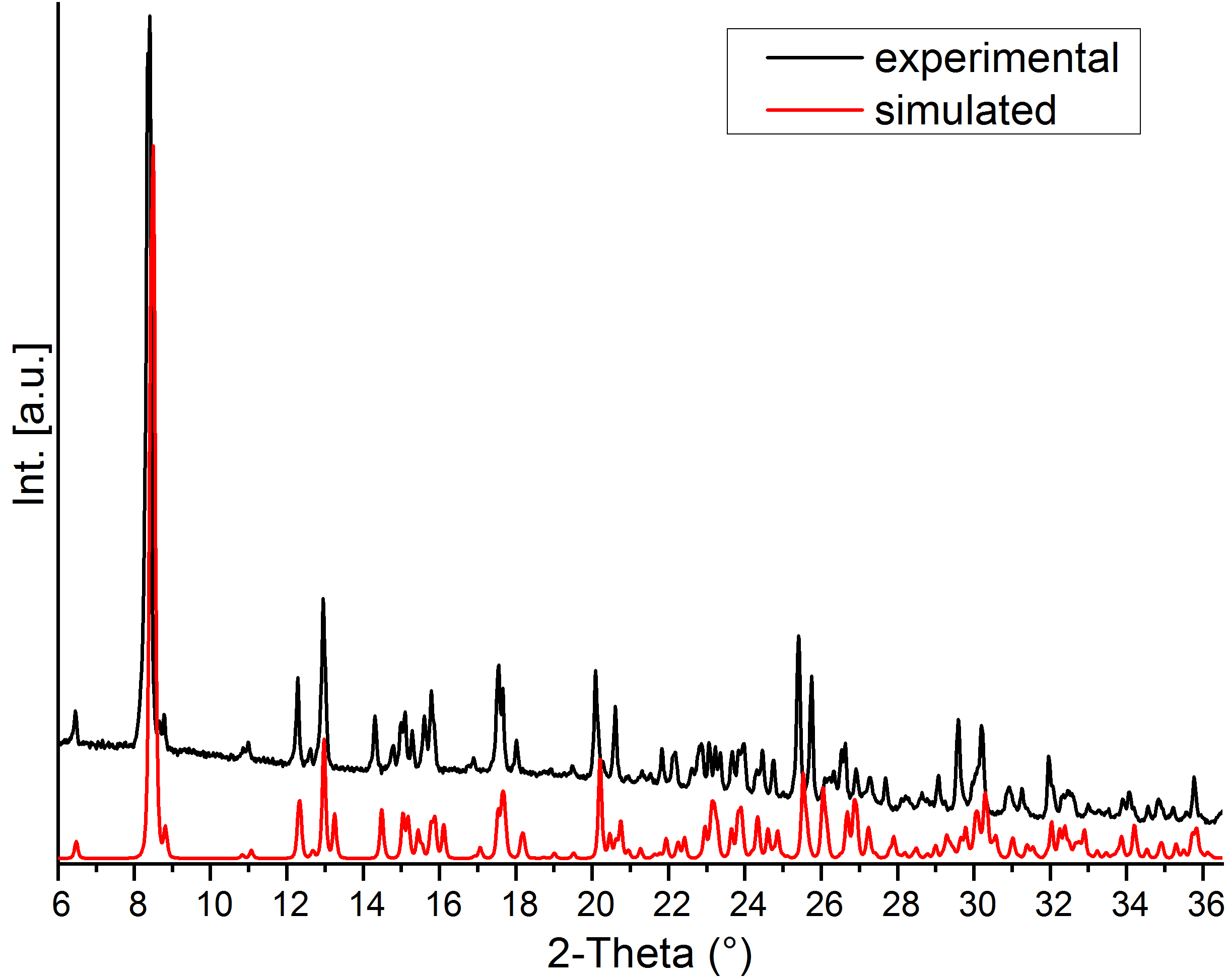

Supplement: Supplementary file 3 [file e-76-00032-sup3.jpg]
